# Supplementary material for: Intestinal parasites may be associated with later behavioral problems in internationally adopted children
Source: PLoS One. 2021 Jan 25;16(1):e0245786. doi: 10.1371/journal.pone.0245786 (PMC7833226; doi:10.1371/journal.pone.0245786)

PLOS ONE

Intestinal parasites associated with later behavioral problems in internationally adopted children

--Manuscript Draft--

**Manuscript Number:**	PONE-D-20-08573
**Article Type:**	Research Article
**Full Title:**	Intestinal parasites associated with later behavioral problems in internationally adopted children
**Short Title:**	Intestinal parasites and later behavioral problems
**Corresponding Author:**	Anna-Riitta Heikkilä, M.D.<br>Helsingin Yliopisto Laaketieteellinen tiedekunta<br>Helsinki, FINLAND
**Keywords:**	internationally adopted children; intestinal parasites; CBCL; FinAdo study
**Abstract:**	Aim: At arrival in new home country, internationally adopted children often have intestinal parasites. International adoptees also exhibit more behavioral problems to a greater extent than their biological peers. We examined whether intestinal parasite infections in international adoptees on arrival are associated with their later behavioral and emotional problems.<br>Methods: Data for this study were sourced from the Finnish Adoption (FinAdo) questionnaire study for all internationally adopted children under 18 years ($n = 1450$) who arrived in Finland from 1985 to 2007. A total of 1293 families provided sufficient information on the adoptee's background, parasitic status on arrival, and behavioral symptoms at the median time of 5 years after arrival (mean age = 7.8 years). Behavioral and emotional disorders were evaluated with the Child Behavior Checklist (CBCL). Statistical analyses were conducted using linear regression.<br>Results: Of the 1293 families, 206 reported intestinal parasites in their adopted children on arrival. Parasite-infected children had subsequently higher CBCL problem scores than the children without parasites ($p < 0.001$).<br>Conclusions: Intestinal parasite infections in early childhood are associated with children's later psychological wellbeing, even in children who move to a country with a low prevalence of parasites.
**Order of Authors:**	Anna-Riitta Heikkilä, M.D.<br>Marko Elovainio<br>Hanna Raaska<br>Jaakko Matomäki<br>Jari Sinkkonen<br>Helena Lapinleimu
**Opposed Reviewers:**	
**Additional Information:**	
**Question**	**Response**
**Financial Disclosure**<br><br>Enter a financial disclosure statement that describes the sources of funding for the work included in this submission. Review the submission guidelines for detailed requirements. View published research articles from *PLOS ONE* for specific examples.	AH: The Foundation of Pediatric Research, Finland (personal grant, no specific grant number), https://www.lastentautientutkimussaatio.fi<br><br>HL: Finland's Slot Machine Association (RAY) (personal grant, no specific grant number), https://www.stea.fi/web/en/frontpage; The Gyllenberg Association (personal grant, no specific grant number), https://gyllenbergs.fi; the Foundation for Pediatric Research, Finland (personal grant, no specific grant number), www.lastentautientutkimussaatio.fi; EVO Grant from Turku University Hospital (personal grant, no specific grant number),

Powered by Editorial Manager® and ProduXion Manager® from Aries Systems Corporation

This statement is required for submission and **will appear in the published article** if the submission is accepted. Please make sure it is accurate.<br><br>**Unfunded studies**<br>Enter: *The author(s) received no specific funding for this work.*<br><br>**Funded studies**<br>Enter a statement with the following details:<br>• Initials of the authors who received each award<br>• Grant numbers awarded to each author<br>• The full name of each funder<br>• URL of each funder website<br>• Did the sponsors or funders play any role in the study design, data collection and analysis, decision to publish, or preparation of the manuscript?<br>• **NO** - Include this sentence at the end of your statement: *The funders had no role in study design, data collection and analysis, decision to publish, or preparation of the manuscript.*<br>• **YES** - Specify the role(s) played.<br><br>* typeset	https://www.utu.fi/fi/tutkimus/tutkimusrahoituksen-tuki; The Tiukula foundation (personal grant, no specific grant number); the Yrjö Jahnsson Association (personal grant, no specific grant number) https://www.yjs.fi;<br>Finnish Cultural Foundation, Varsinais-Suomi Regional fund (personal grant, no specific grant number) https://skr.fi/en<br><br>JS: The Tiukula Foundation (personal grant, no specific grant number); Finland's Slot Machine Association (RAY) (personal grant, no specific grant number), https://www.stea.fi/web/en/frontpage<br><br>The funders had no role in study design, data collection and analysis, decision to publish, or preparation of the manuscript.
**Competing Interests**<br><br>Use the instructions below to enter a competing interest statement for this submission. On behalf of all authors, disclose any competing interests that could be perceived to bias this work—acknowledging all financial support and any other relevant financial or non-financial competing interests.<br><br>This statement **will appear in the published article** if the submission is accepted. Please make sure it is accurate. View published research articles from *PLOS ONE* for specific examples.	The authors have declared that no competing interests exist.

**NO authors have competing interests**<br><br>Enter: *The authors have declared that no competing interests exist.*<br><br>**Authors with competing interests**<br><br>Enter competing interest details beginning with this statement:<br><br>*I have read the journal's policy and the authors of this manuscript have the following competing interests: [insert competing interests here]*<br><br>* typeset	
**Ethics Statement**<br><br>Enter an ethics statement for this submission. This statement is required if the study involved:<br><br>• Human participants<br>• Human specimens or tissue<br>• Vertebrate animals or cephalopods<br>• Vertebrate embryos or tissues<br>• Field research<br><br>Write "N/A" if the submission does not require an ethics statement.<br><br>General guidance is provided below. Consult the submission guidelines for detailed instructions. **Make sure that all information entered here is included in the Methods section of the manuscript.**	The study was approved by the Ethics Review Committee of the Hospital District of Southwest Finland. The approval number: ETMK102/180/2012, the statement 11-20-2012 §317.

Powered by Editorial Manager® and ProduXion Manager® from Aries Systems Corporation

**Format for specific study types**<br><br>**Human Subject Research (involving human participants and/or tissue)**<br>• Give the name of the institutional review board or ethics committee that approved the study<br>• Include the approval number and/or a statement indicating approval of this research<br>• Indicate the form of consent obtained (written/oral) or the reason that consent was not obtained (e.g. the data were analyzed anonymously)<br><br>**Animal Research (involving vertebrate animals, embryos or tissues)**<br>• Provide the name of the Institutional Animal Care and Use Committee (IACUC) or other relevant ethics board that reviewed the study protocol, and indicate whether they approved this research or granted a formal waiver of ethical approval<br>• Include an approval number if one was obtained<br>• If the study involved *non-human primates*, add *additional details* about animal welfare and steps taken to ameliorate suffering<br>• If anesthesia, euthanasia, or any kind of animal sacrifice is part of the study, include briefly which substances and/or methods were applied<br><br>**Field Research**<br><br>Include the following details if this study involves the collection of plant, animal, or other materials from a natural setting:<br>• Field permit number<br>• Name of the institution or relevant body that granted permission	
**Data Availability**<br><br>Authors are required to make all data underlying the findings described fully available, without restriction, and from the time of publication. PLOS allows rare exceptions to address legal and ethical concerns. See the PLOS Data Policy and FAQ for detailed information.	No - some restrictions will apply

A Data Availability Statement describing where the data can be found is required at submission. Your answers to this question constitute the Data Availability Statement and **will be published in the article**, if accepted.<br><br>**Important:** Stating 'data available on request from the author' is not sufficient. If your data are only available upon request, select 'No' for the first question and explain your exceptional situation in the text box.<br><br>Do the authors confirm that all data underlying the findings described in their manuscript are fully available without restriction?	
**Describe where the data may be found in full sentences. If you are copying our sample text, replace any instances of XXX with the appropriate details.**<br><br>• If the data are **held or will be held in a public repository**, include URLs, accession numbers or DOIs. If this information will only be available after acceptance, indicate this by ticking the box below. For example: *All XXX files are available from the XXX database (accession number(s) XXX, XXX.*).<br>• If the data are all contained **within the manuscript and/or Supporting Information files**, enter the following: *All relevant data are within the manuscript and its Supporting Information files.*<br>• If neither of these applies but you are able to provide **details of access elsewhere**, with or without limitations, please do so. For example:<br><br>*Data cannot be shared publicly because of [XXX]. Data are available from the XXX Institutional Data Access / Ethics Committee (contact via XXX) for researchers who meet the criteria for access to confidential data.*<br><br>*The data underlying the results presented in the study are available from (include the name of the third party*	Data cannot be shared publicly because of GDPR, local protection act.

and contact information or URL).<br>• This text is appropriate if the data are owned by a third party and authors do not have permission to share the data.<br><br>* typeset	
Additional data availability information:	Tick here if the URLs/accession numbers/DOIs will be available only after acceptance of the manuscript for publication so that we can ensure their inclusion before publication.

Powered by Editorial Manager® and ProduXion Manager® from Aries Systems Corporation

Dear Editor-in-Chief,

I wish to submit the digital version of our manuscript entitled “Intestinal parasites associated with later behavioral problems in internationally adopted children.” I hope you will find this research article interesting enough to be considered for publication in *PLOS One.*

Many recent studies have shown that intestinal parasites are common among internationally adopted children at the time of arrival in their new home country. Internationally adopted children also often display behavioral symptoms after arrival and later in their life. In this study, we assessed whether intestinal parasitic infections in early childhood are somehow associated with psychiatric symptoms later in the lives of the infected children. To the best of our knowledge, this research study is the first one to discover such an association even when the studied internationally adopted children move to the new country with a low prevalence of intestinal parasites and parasitic infections. The reason behind this association is not known. We suggest that these results may be attributed to intestinal parasites modulating intestinal microbiota and thereby impacting the children’s developing brains through the microbiota-gut-brain axis.

On behalf of all the authors, I affirm that this manuscript is being submitted only to *PLOS One*. Moreover, this manuscript will not be submitted elsewhere while under consideration and it has not been published elsewhere. Should it be published in *PLOS One*, it will not be published elsewhere, either in similar form or verbatim, without the permission of the editors. These restrictions do not apply to abstracts or to press reports of presentations at scientific meetings.

All the authors are responsible for the reported research. All the authors have participated in the concept and design, analysis and interpretation of data, and drafting or revising of the manuscript. The submitted manuscript has been approved by all the authors. None of the authors have had prior interactions with *PLOS One* regarding this manuscript. The authors have no conflicts to declare.

We request that either Academic Editor, Francesca Lembo or Marie Lof, handle our manuscript. We do not oppose any potential reviewers.

I look forward to hearing from you.

Sincerely,

Anna-Riitta Heikkilä, MD
Department of Pediatrics, University of Helsinki
P.O. Box 22, 00014 University of Helsinki, Finland,
email: anna-riitta.heikkila@helsinki.fi
Tel: +3580504093232
Fax: +358947175299

Manuscript

Click here to download Manuscript Intestinal parasites and later behavioral problems.docx

Intestinal parasites associated with later behavioral problems in internationally adopted children

Anna-Riitta Heikkilä[1*], Marko Elovainio[2, 3], Hanna Raaska[4], Jaakko Matomäki[5], Jari Sinkkonen[6], and Helena Lapinleimu[7]

[1]Department of Pediatrics, University of Helsinki and Helsinki University Central Hospital, Helsinki, Finland

[2]Research Program Unit, Faculty of Medicine, University of Helsinki, Helsinki, Finland

[3]Finnish Institute for Health and Welfare, Helsinki, Finland

[4]Department of Child Psychiatry, Helsinki University Central Hospital, Helsinki, Finland

[5]Clinical Research Centre, Turku University Hospital, Turku, Finland

[6]Department of Child Psychiatry, University of Turku, Turku, Finland

[7]Department of Pediatrics and Adolescent Medicine, University of Turku and Turku University Hospital, Turku, Finland

*Corresponding author

E-mail: anna-riitta.heikkila@helsinki.fi (AH)

The all authors contributed equally to this work.

Abstract

Aim: At arrival in new home country, internationally adopted children often have intestinal parasites. International adoptees also exhibit more behavioral problems to a greater extent than their biological peers. We examined whether intestinal parasite infections in international adoptees on arrival are associated with their later behavioral and emotional problems.

Methods: Data for this study were sourced from the Finnish Adoption (FinAdo) questionnaire study for all internationally adopted children under 18 years (n = 1450) who arrived in Finland from 1985 to 2007. A total of 1293 families provided sufficient information on the adoptee's background, parasitic status on arrival, and behavioral symptoms at the median time of 5 years after arrival (mean age = 7.8 years). Behavioral and emotional disorders were evaluated with the Child Behavior Checklist (CBCL). Statistical analyses were conducted using linear regression.

Results: Of the 1293 families, 206 reported intestinal parasites in their adopted children on arrival. Parasite-infected children had subsequently higher CBCL problem scores than the children without parasites ($p < 0.001$).

Conclusions: Intestinal parasite infections in early childhood are associated with children's later psychological wellbeing, even in children who move to a country with a low prevalence of parasites.

Introduction

Internationally adopted children often show growth and developmental delays on arrival in their new home country (1-6). Many studies have also shown international adoptees to have more behavioral problems compared to their same-age, non-adopted peers (7, 8). Although many psychological explanations exist for growth and developmental delays also more physiological explanations are plausible. Intestinal parasite infections are common on arrival among internationally adopted children, especially among adoptees from countries with poor living conditions and subpar sanitation (3, 4, 6, 9-16). Intestinal parasites in children are associated with growth and developmental delays (17-19) as well as micronutrient deficiencies (20). Micro-nutritional deficiencies, such as iron deficiency, are known to be associated with multiple developmental problems (21-23). Developmental problems, in turn, have been shown to cause increased behavioral problems (24).

Intestinal parasites also affect the microbial imbalance of intestinal microbiota (25-27). Intestinal parasites alter the intestinal physiology and may impact on the microbiota-gut-brain axis (27-29). The gut microbiome affects brain function by changing immunological mechanisms, causing inflammation, and modifying the functions of several neurotransmitters (30-34). Recent studies have also suggested that the intestinal microbiome plays a role in neurodevelopmental and neurobehavioral conditions (30), and some clues exist about their association with personality traits (31) and psychological symptoms (34).

No previous studies have shown whether the risks associated with earlier intestinal parasite infections persist after the child moves to more nurturing living conditions, when the intestinal parasitic infection has been efficiently treated, and when

the child has had no reinfections. Therefore, in this study we investigated whether an association exist between intestinal parasites and later behavioral problems among internationally adopted children.

Methods

Participants

This work is part of the FINnish ADOption (FinAdo) study, which includes all children internationally adopted through legalized adoption organizations in Finland between 1985 and 2007. The survey data were collected through questionnaires between December 2007 and March 2009, and included two mailed questionnaires for non-respondents. The questionnaires covered child-related factors, family-related factors, background data of the adoptive parents as well as the information of intestinal parasites of adopted children on arrival in Finland. As a part of the survey, the parents also completed the Child Behavior Checklist (CBCL) questionnaire (7, 8) on the behavioral and emotional status of their children at the mean age of 7.8 (*SD* = 4.3) years, and the median age of 7 years. The median time after adoption in Finland was 5 years during the time the survey was conducted.

The original cohort included 1450 international adoptees (816 girls, 56%) under 18 years old (mean age = 7.5 years, *SD* = 4.4) at the commencement of study. The participation rate for the study was 55.7% (35). The final sample comprises a total of 1293 under the age of 18 years (724 girls, 56%) who had been screened for the intestinal

parasites and whose adoptive parents provided sufficient information about their behavioral characteristics during the evaluation (Fig 1).

Fig 1. Selection of the study population.

The study was approved by the Ethics Review Committee of the Hospital District of Southwest Finland, and the participating children and their parents provided their written consent to participate.

Measures

Child-related factors

The child's gender, age at adoption, age at evaluation, and country of birth were reported by the parents. The type and number of placements before adoption were classified into three categories for statistical analysis. The place of origin was categorized by continent and region. Parents were also asked about any medical diagnoses their children received from Finnish medical evaluations soon after arrival or later.

Growth values of adopted children at arrival were reported in the parental questionnaire. For the analysis growth values were expressed as *z*-scores, which were compared against the growth charts of the World Health Organization (36). Weight-for-age, height-for-age, and head circumference-for-age were used separately for both genders. The *z*-scores ranged from -6 *SD* to +3 *SD* for weight, from -6 *SD* to +2.5 *SD* for height, and from -5 *SD* to +3 *SD* for head circumference. Children with missing growth values were excluded from the fourth step of the linear regression analysis. *z*-scores below -2 *SD* in each category were considered to indicate delayed growth.

Parasitic status

In Finland internationally adopted children undergo health and medical evaluations in public health care centers soon after arrival in the country. All the children were recommended tests for parasite infections by means of stool examinations for ova and parasites (2 specimens taken 24 to 48 hours apart) at the local health care centers. The parents were requested to obtain their children's test results from the health care centers and attach them with the completed questionnaire report. Thus, all the medical data were collected retrospectively from the parents via the questionnaire.

Family-related factors

In addition, the parents answered a series of questions about their own marital and socioeconomic status and health. Each adoptive family's socioeconomic status was classified into one of four categories according to the vocation of the family member with the highest status. The marital status of the each parent was classified into one of two categories.

Behavioral problems

The CBCL was chosen to measure the adoptees' behavioral and emotional problems because of its wide use in adoption research (7, 8), as well as the fact it has shown good psychometric reliability in studies on internationally adopted (8). The CBCL provides a total score for behavioral problems and also separates the scores for internalizing and externalizing behavioral symptoms. Internalizing behavioral symptoms reflect problems mainly within the self, such as anxiety, depression, somatic complaints without medical cause, and withdrawal from social contacts. Externalizing behavioral signs include conflict with others, such as rule-breaking or aggressive behavior (37). Each item

was rated as 0 (not true), 1 (somewhat or sometimes true), and 2 (very true or often true). The higher the child's scores in the CBCL, the greater the extent of his/her behavioral problems.

In this study we used two CBCL questionnaires: the CBCL for ages 1.5 to 5 years with 100 questions and that for ages 6 to 18 years with 113 questions (8). These questionnaires produce identical dimensions and total scores, thus providing comparable age-adjusted scores. Completed CBCL questionnaires were received for 1302 of the 1450 cases.

Statistical analyses

Delete. To read Data analyses

Statistical analyses were performed with the STATA program package (version 12), and *p* values below 0.05 were considered statistically significant. All the significance tests were two-tailed. The child's current age, age on arrival, and CBCL scale were analyzed as continuous variables. Gender, country of origin, placement before adoption, the child's disabilities, presence of anemia (Hb limit < 11.5 g/dL), socioeconomic status of the adoptive family, and the adoptive parent's marital status were used as the categorical variables.

Correct to read "Data obtained were analysed using STATA program package ......

Analyses were done using linear regression in four steps. The first step tested for any association between the parasites and behavioral problems. In the second step, the association was adjusted for child-related factors. In the third step, the association was additionally adjusted for family-related factors. The final model also included information on anemia and the child's growth variables at adoption. For the sensitivity evaluation, we also assessed the interaction effect between factors related to the child's background and intestinal parasite infection on the scales provided by the CBCL.

Further analyses

delete we also assessed, but to read " assessment on the interacting effects between .....

Results

Of the 1293 children screened, 206 had at least one intestinal parasite on arrival in Finland (Fig 1), with *Giardia lamblia* being the most prevalent. Of the 206 children, 122 (59.2%) had *Giardia* in their stool samples, which collected after arrival, and 62 children (30.1%) had no species listed at all. Of the children, 13 (6.3%) had *Entamoeba histolytica*, 7 (3.4%) had *Blastocystis hominis*, 5 (2.4%) had tapeworms, 4 (1.9%) had strongyloides, and 2 (1.0%) had *Hymenolepsis nana*. Only 2 (1.0%) cases involved pinworms (*Enterobius vermicularis*), and 17 (8.3%) families omitted species of the worm found. Single cases emerged of *Ascaris lumbricoides*, Whipworm, or Schistosomiasis. Of the adoptees with intestinal parasitism, 7.8% had multiple intestinal parasites on arrival, but because of this number was low, the influence of multiple parasites was not analyzed separately.

The children with a detectable parasite infection at adoption were older (mean age = 2.6 years) than were those without parasites (mean age = 2.1 years; p = 0.002) (Table 1). At the time of the evaluation, the mean ages of the children with a parasite infection at arrival was 8.2 years, and for uninfected children, 7.7 years. Of the 206 adopted children with intestinal parasites on arrival, 51.9% were from Asia, 26.7% from Africa, 9.7% from South America, and 11.7% from Eastern Europe. The number and type of pre-adoption placements had no association with the prevalence of intestinal parasite infections. In terms of the growth values, the presence of an intestinal parasite infection was associated only with sub-standard height (p <0.001) (Table 1).

Table 1. Characteristics of the FinAdo study sample presented as means (standard deviation) and numbers (percentage of the sample)

**Characteristic**	All	Children with parasites	Children with no parasites	*p* value

	n (%)/Mean (*SD*)	*n* (%)	Mean (*SD*)	*n* (%)	Mean (*SD*)	
**Children**	1293 (100)	206 (15.9)		1087 (84.1)		
Girls	724 (56.0)	103 (14.2)		621 (85.8)		0.059
Boys	569 (44.0)	103 (18.1)		466 (81.9)		
**Age on arrival in Finland, years**	2.2 (1.9)		2.6 (1.6)		2.2 (1.9)	0.002
**Age at evaluation, years**	7.8 (4.3)		8.2 (4.4)		7.7 (4.3)	0.154
**Disabilities***	137 (10.6)	16 (11.7)		121 (88.3)		0.145
**Anemia**						0.86
No	1174 (90.1)	189 (16.1)		985 (83.9)		
Yes	110 (8.5)	17 (15.5)		93 (84.5)		
**Growth on arrival in Finland**						
Weight (*SD*)	1099	184	-0.9 (1.2)	915	-0.8 (1.1)	0.139
Height (*SD*)	993	165	-1.8 (1.3)	828	-1.2 (1.3)	<0.001
Head circumference (*SD*)	765	108	-0.4 (1.0)	657	-0.4 (1.1)	0.923
**Continent of birth**						<0.001
Asia	712 (55.1)	107 (15.0)		605 (85.0)		
Africa	141 (10.9)	55 (39.0)		86 (61.0)		
South America	179 (13.8)	20 (11.2)		159 (88.8)		
Eastern Europe	261 (20.2)	24 (9.2)		237 (90.8)		
**Number and type of placements before adoption**						0.928
Foster home	114 (8.8)	19 (16.7)		95 (83.3)		
Orphanage	717 (55.6)	116 (16.2)		601 (83.8)		
Many placements	459 (35.6)	71 (15.5)		388 (84.5)		
**Parents**						
**Adoptive family's socioeconomic status**						0.605
Upper middle class	747 (59)	122 (16.3)		625 (83.7)		
Lower middle class	261 (20.6)	40 (15.3)		221 (84.7)		
Working class	224 (17.7)	33 (14.7)		191 (85.3)		
Other	34 (2.7)	8 (23.5)		26 (76.5)		
**Adoptive parent's marital status**						0.133
Married parents	1100 (86.5)	169 (15.4)		931 (84.6)		
Single-parent household	171 (13.5)	34 (19.9)		137 (80.1)		

*Deafness or impaired hearing in both ears, blindness or visual defect in both eyes, mental retardation or intellectual disability, and autism

The adopted children with an intestinal parasite infection on arrival had significantly higher CBCL problem scores than their uninfected counterparts for all three categories: total problem scores ($p < 0.001$), externalizing problem scores ($p = 0.002$), and internalizing problem scores ($p = 0.003$). Addition of child- or family-related factors did not affect the association. Among the intestinal parasite-infected adopted children, growth

delays or anemia at adoption were not associated with higher CBCL problem scores for any child (Table 2). No significant interaction effects emerged between parasite infection and gender (p = 0.80–0.89), age at adoption (p = 0.22–0.90), or socioeconomic position (p = 0.26–0.54) in any of the CBCL symptom categories. Interaction effects occurred only between age and parasite infection for internalizing symptoms (p = 0.045). Among children adopted from South America, intestinal parasite infections raised the problem scores in all the CBCL categories to a greater extent in the other cases (p for interactions = 0.018–0.001). The adopted children from Africa, (unstandardized regression coefficient or b = 1.6, p = 0.04), South-America (b = 2.32, p < 0.01), and Eastern Europe (b = 5.07, p < 0.001) had more CBCL externalizing symptoms than the children from Asia. The children adopted from Eastern Europe also had more internalizing symptoms than the children adopted from Asia (b = 1.67, p < 0.001). The children adopted from South Africa (b = 4.1, p = 0.024) and from Eastern Europe (b = 14.6, p < 0.001) had higher total CBCL problem scores than those from Asia.

Table 2. Associations between parasite status and the CBCL symptoms among international adoptees (*n* = 1293 - 1069). (The figures are standardized regression coefficients (*B*), unstandardized regression coefficients (*b*), standard errors (*SE*) and *p* values.)

	Step 1, *n* = 1293			Step 2, *n* = 1285			Step 3, *n* = 1259			Step 4, *n* = 1069		
	**Externalizing problems** *B/b (SE) p*	**Internalizing problems** *B/b (SE) p*	**CBCL total score** *B/b (SE) p*	**Externalizing problems** *B/b (SE) p*	**Internalizing problems** *B/b (SE) p*	**CBCL total score** *B/b (SE) p*	**Externalizing problems** *B/b (SE) p*	**Internalizing problems** *B/b (SE) p*	**CBCL total score** *B/b (SE) p*	**Externalizing problems** *B/b (SE) p*	**Internalizing problems** *B/b (SE) p*	**CBCL total score** *B/b (SE) p*
**Parasite infection on arrival**	0.09/2.19 (0.70) 0.002	0.08/1.28 (0.43) 0.003	0.09/5.41 (1.69) 0.001	0.09/2.35 (0.68) 0.001	0.10/1.47 (0.43) 0.001	0.10/6.01 (1.57) < 0.001	0.09/2.20 (0.67) 0.001	0.09/1.40 (0.43) 0.001	0.10/5.81 (1.59) < 0.001	0.09/2.24 (0.68) 0.001	0.10/1.45 (0.43) 0.001	0.10/5.94 (1.59) < 0.001
**Age at the time of the questionnaire study**				-0.12/-0.25 (0.06) < 0.001	-0.00/-0.00 (0.04) 0.971	-0.05/-0.28 (0.15) 0.063	-0.11/-0.24 (0.07) < 0.001	0.01/0.01 (0.04) 0.766	-0.04/-0.23 (0.16) 0.138	-0.11/-0.25 (0.07) < 0.001	0.00/0.01 (0.04) 0.880	-0.05/-0.25 (0.16) 0.106
**Age on arrival in Finland**				0.11/0.56 (0.16) 0.001	0.02/0.07 (0.10) 0.467	0.09/1.14 (0.36) 0.002	0.11/0.49 (0.16) 0.002	0.02/0.06 (0.10) 0.525	0.09/1.07 (0.37) 0.004	0.10/0.49 (0.16) 0.002	0.02/0.06 (0.10) 0.523	0.09/1.07 (0.37) 0.004
**Gender**				0.14/2.66 (0.51) < 0.001	-0.03/-0.33 (0.32) 0.306	0.10/4.41 (1.19) < 0.001	0.15/2.71 (0.51) < 0.001	-0.03/-0.36 (0.33) 0.274	0.10/4.41 (1.21) < 0.001	0.14/2.68 (0.52) < 0.001	-0.04/-0.41 (0.33) 0.215	0.10/4.28 (1.22) < 0.001
**Continent of birth**				0.12/0.90 (0.22) < 0.001	0.04/0.17 (0.14) 0.252	0.12/2.18 (0.53) < 0.001	0.12/0.90 (0.23) < 0.001	0.04/0.18 (0.15) 0.233	0.12/2.24 (0.54) < 0.001	0.12/0.92 (0.23) < 0.001	0.04/0.20 (0.15) 0.172	0.13/2.31 (0.54) < 0.001
**Number and type of pre-adoption placements**				0.01/0.03 (0.10) 0.788	0.04/0.09 (0.06) 0.163	0.02/0.17 (0.24) 0.488	0.01/0.02 (0.10) 0.855	0.04/0.09 (0.07) 0.170	0.02/0.14 (0.24) 0.550	0.00/0.02 (0.10) 0.859	0.04/0.09 (0.07) 0.163	0.02/0.15 (0.24) 0.534
**Disabilities**				0.22/6.68 (0.82) < 0.001	0.23/4.36 (0.53) < 0.001	0.32/23.3 (1.93) < 0.001	0.22/6.46 (0.83) < 0.001	0.23/4.33 (0.53) < 0.001	0.31/22.8 (1.96) < 0.001	0.22/6.47 (0.82) < 0.001	0.23/4.34 (0.53) < 0.001	0. 32/22.9 (1.95) < 0.001
**Adoptive family's socioeconomic status**							0.01/0.11 (0.28) 0.711	0.03/0.20 (0.18) 0.268	0.03/0.68 (0.67) 0.312	0.01/0.13 (0.28) 0.657	0.03/0.21 (0.18) 0.245	0.03/0.72 (0.67) 0.282
**Adoptive parent's marital status**							-0.01/-0.12 (0.46) 0.795	-0.02/-0.24 (0.29) 0.414	-0.02/-0.85 (1.08) 0.433	-0.01/-0.13 (0.46) 0.782	-0.02/-0.24 (0.29) 0.429	-0.02/-0.85 (1.08) 0.429
**Existence of anemia**										-0.03/-1.14 (0.88) 0.195	-0.00/-0.10 (0.56) 0.855	-0.03/-2.27 (2.07) 0.272
**Growth in terms of weight**										-0.05/-0.38 (0.40) 0.341	-0.09/-0.41 (0.27) 0.133	-0.03/-0.55 (0.97) 0.571
R^2	0.01	0.01	0.01	0.13	0.07	0.18	0.13	0.08	0.18	0.13	0.08	0.18

Discussion

In this study, we found that intestinal parasite infection upon entry to the country of adoption was associated with higher CBCL problem scores later in life (at the median time of 5 years after adoption). The reason behind this association is not known. Intestinal parasite infections may cause behavioral problems via multiple plausible biological mechanisms. Intestinal parasites may influence the maturing and developing brain by causing micronutrient deficiencies, or by affecting gut-brain-axis by changing the intestinal microbiome. Changes in the intestinal microbiota after antibiotic treatment for intestinal parasite infections may also impact the children's brain. In any case, our results suggest that intestinal parasites are harmful in the early years of life to the internationally adopted children, who are undergoing rapid growth and development of the brain. These years of life are also considered to be a considerably important time for the maturation of the gut microbiome and the immune system (38).

Intestinal parasitic infections may disturb intestinal absorption processes. Although the presence of anemia could not to explain the association between intestinal parasite infections and later behavioral problems (Table 2), it is possible that intestinal parasitic infections contribute to later behavioral symptoms through micronutrient deficiencies, such as iron deficiency without anemia (20). Fuglestad and coauthors (2008) showed that iron deficiency among internationally adopted children was more difficult to remedy than among adopted children with intestinal Giardia parasitism at the time of arrival. Later, this group (2013) showed that children with iron deficiency at adoption or 6 months later were more likely to score below average on tests of learning capacity 6 to 7 months post-adoption, and only 20% of the children with iron deficiency were anemic. Iron deficiency also has an adverse impact on neurobehavioral predictability for months after

adoption and exhibits long-lasting effects on children's development (21-23). Delays in cognitive development and problems in executive functions have repeatedly been shown to predict behavioral problems (39, 40). Thus, delays in cognitive development may also represent a physiological mechanism linking the nutrient deficiencies caused by intestinal parasites to later behavioral problems also in the adopted children.

As mentioned previously, the other plausible mechanism behind the association between intestinal parasite infection and later behavioral symptoms involves changes in intestinal microbiota caused by intestinal parasite infections. Intestinal parasites are shown to cause imbalance in microbiota and alterations to the intestinal microbiome by modulating the intestinal microbiota (27-29). Although research in this area is still limited, some reports indicate that gut microbiota may be associated with neuropsychiatric and neurodevelopmental conditions (30) and even adulthood personality traits (31). Further, microbial dysbiosis in adults has been associated with later anxiety (32) or depression-related behaviors (33). In their pre-adoptive surroundings, the internationally adopted children have been already exposed early in life to alterations caused by intestinal parasites on their intestinal microbiome, and this early exposure also impacts the developing brain through gut-brain axis.

The use of antibiotics is also an alternative explanatory mechanism between the association with intestinal parasitic infections and later behavioral problems in our study population. Intestinal parasitic infections are often treated with antibiotics, and the use of antibiotics in the early years of life is known to have a long-lasting influence on gut microbiota, the intestinal microbiome, and overall health (41, 42). Recent studies have also shown an association between antibiotic treatment in infancy and later neurodevelopmental difficulties, such as behavioral problems (43, 44).

The prevalence of intestinal parasites among internationally adopted children on arrival in Finland was 15.6%, which is comparable to the prevalence in adopted children in earlier studies in the other countries (3, 9-11, 45) but lower than the reported in some recent works (6, 12-15). This difference may be attributed to the development of better stool screening technologies, such as DNA-based methods, or reporting biases. The continent of origin of the adopted children was associated with the prevalence of parasites on arrival (Table 1). Similar to already reported data (3, 4, 6, 9-15, 45), the most prevalent pathogenic intestinal parasite detected among the adopted children was *Giardia lamblia*, but the parents were unable to specify the species of the parasites or worms in 35% of the cases. Public health care is free for all residents in Finland, and the first medical examinations of adopted children usually occur in public health centers supervised by medical professionals according to the recommendations of the Finnish adoption organizations. These guidelines are comparable with the screening protocols of newly arrived adopted children internationally (46). Thus it was assumed that any intestinal parasites found during the medical screenings were adequately treated. However, this study could not record such actual treatments. Moreover, we are unaware if any children had been treated for the parasite infections in their country of origin prior to travelling to Finland.

In this study, the CBCL questionnaire was considered an appropriate indicator of a child’s later psychological symptoms, since it is a widely used, valid, and well-known method to evaluate children’s behavioral and emotional problems, and is also considered suitable for questionnaire-based studies (8). The grouping used here (internalizing, externalizing, and total score) is commonly applied in other research (7). In a former meta-analysis, the behavior problems of internationally adopted children were measured using the CBCL, and the related measures showed only small differences between the

outcomes of the adopted children and non-adopted, parent-reared children (7). In this study, the behavior of the internationally adopted children was evaluated using the CBCL questionnaire at the time of evaluation, approximately 5 years after adoption. This finding also underscores the importance brain development in the early years regardless of the influence mechanism of the intestinal parasites.

This study uncovered associations between an earlier intestinal parasite infection and higher CBCL problem scores in all three CBCL categories (externalizing problems, internalizing problems and CBCL total scores). The stronger association observed in the older children may be due to their longer exposures to parasite infections. The association between the CBCL symptoms and the parasite infection in the children adopted from South America may be due to the differences in the local parasites or the different risk factors, such as being exposed to drugs in utero (4).

This study was partly retrospective, as the health of the adopted children upon their arrival in Finland was ascertained from their parents' responses to questions. The time delay from the arrival date to these responses (approximately 5 years) may have resulted in a bias. In general, however, the adoptive parents answered the questions precisely and had good knowledge of the health and medical issues of their adopted children because they had undergone substantial training before the adoption. This study also tried to ascertain whether the studied children were anemic or had iron deficiency on arrival, but doing so was not possible as their iron status was not registered.

Earlier international studies using standardized measures such as the CBCL to evaluate children coming from different continents suggest that no significant differences exist in the extent or types levels of symptoms (47). Thus, the differences in "normality" in behavior between various countries are rather small and difficult to evaluate.

Similar to other studies, we used the CBCL as a continuous measure for the two border groups and the total score (7). However, we were unable to determine specific cut-off points for clinically relevant behavioral problems from these scores. However, such measures have been shown to predict clinical outcomes.

Conclusion

Our results suggest of a five-year follow up post-adoption suggested that intestinal parasite infections among internationally adopted children are associated with higher behavioral problem scores. The association persisted even after these children moved to a country with a low prevalence of parasites. The effect sizes of intestinal parasite infections on behavioral problems were relatively small. Behavioral problems are obviously multifactorial, and the finding that intestinal parasite infections can be strong predictors of behavioral problems at some point later in life was unexpected. However, the association between intestinal parasites and later behavioral problems was stronger than that between intestinal parasites and any other factors measured in this study, except disability. This suggests that internationally adopted children should be screened for intestinal parasites, and intestinal parasitism should be appropriately treated soon after arrival, especially among these at-risk populations, as the children are typically undergoing rapid growth and brain development at that age.

Acknowledgements

The authors are thankful to the all the internationally adopted children and their parents for the participation in this study.

Conflict of interest

The authors have no conflicts of interest to declare.

References

1. Weitzman C, Albers L. Long-term developmental, behavioral, and attachment outcomes after international adoption. Pediatric clinics of North America. 2005;52(5):1395-419, viii. doi:10.1016/j.pcl.2005.06.009
2. Cohen NJ, Lojkasek M, Zadeh ZY, Pugliese M, Kiefer H. Children adopted from China: a prospective study of their growth and development. Journal of child psychology and psychiatry, and allied disciplines. 2008;49(4):458-68. doi:10.1111/j.1469-7610.2007.01853.x
3. Miller LC, Hendrie NW. Health of children adopted from China. Pediatrics. 2000;105(6):E76.
4. Miller L, Chan W, Comfort K, Tirella L. Health of children adopted from Guatemala: comparison of orphanage and foster care. Pediatrics. 2005;115(6):e710-7. doi:10.1542/peds.2004-2359
5. Fuglestad AJ, Kroupina MG, Johnson DE, Georgieff MK. Micronutrient status and neurodevelopment in internationally adopted children. Acta paediatrica (Oslo, Norway : 1992). 2016;105(2):e67-76. doi:10.1111/apa.13234
6. Van Kesteren L, Wojciechowski M. International adoption from Ethiopia: An overview of the health status at arrival in Belgium. Acta clinica Belgica. 2017;72(5):300-5. doi:10.1080/17843286.2016.1258178
7. Juffer F, van Ijzendoorn MH. Behavior problems and mental health referrals of international adoptees: a meta-analysis. Jama. 2005;293(20):2501-15. doi:10.1001/jama.293.20.2501
8. Hawk B, McCall RB. CBCL behavior problems of post-institutionalized international adoptees. Clinical child and family psychology review. 2010;13(2):199-211. doi:10.1007/s10567-010-0068-x
9. Miller LC, Tseng B, Tirella LG, Chan W, Feig E. Health of children adopted from Ethiopia. Maternal and child health journal. 2008;12(5):599-605. doi:10.1007/s10995-007-0274-4
10. Saiman L, Aronson J, Zhou J, Gomez-Duarte C, Gabriel PS, Alonso M, et al. Prevalence of infectious diseases among internationally adopted children. Pediatrics. 2001;108(3):608-12.
11. Staat MA, Rice M, Donauer S, Mukkada S, Holloway M, Cassedy A, et al. Intestinal parasite screening in internationally adopted children: importance of multiple stool specimens. Pediatrics. 2011;128(3):e613-22. doi:10.1542/peds.2010-3032
12. Totaro C, Bortone B, Putignano P, Sollai S, Galli L, de Martino M, et al. Internationally adopted children: not only infectious diseases! Journal of travel medicine. 2018;25(1). doi:10.1093/jtm/tay036
13. Buonsenso D, Graffeo R, Scarlato L, Acampora A, Grotti G, Scarlato L, et al. Intestinal Parasitic Infections in Internationally Adopted Children: A 10-Year Retrospective Study. The Pediatric infectious disease journal. 2019;38(10):983-9. doi:10.1097/inf.0000000000002399
14. Chiappini E, Zaffaroni M, Bianconi M, Veneruso G, Grasso N, Garazzino S, et al. Italian multicentre study found infectious and vaccine-preventable diseases in children adopted from Africa and recommends prompt medical screening. Acta paediatrica (Oslo, Norway : 1992). 2018. doi:10.1111/apa.14237
15. Nielsen AV, Kjaergaard J, Pedersen TM, Helmuth IG, Nygaard U, Nielsen HW, et al. Infectious diseases detected by screening after arrival to Denmark in internationally adopted children. Acta paediatrica (Oslo, Norway : 1992). 2019. doi:10.1111/apa.15045
16. Henaff F, Hazart I, Picherot G, Baque F, Gras-Le Guen C, Launay E. Frequency and characteristics of infectious diseases in internationally adopted children: a retrospective study in Nantes from 2010 to 2012. Journal of travel medicine. 2015;22(3):179-85. doi:10.1111/jtm.12196
17. Simsek Z, Zeyrek FY, Kurcer MA. Effect of Giardia infection on growth and psychomotor development of children aged 0-5 years. Journal of tropical pediatrics. 2004;50(2):90-3.

18. Miller LC. International adoption: infectious diseases issues. Clinical infectious diseases : an official publication of the Infectious Diseases Society of America. 2005;40(2):286-93. doi:10.1086/427109
19. Oliveira D, Ferreira FS, Atouguia J, Fortes F, Guerra A, Centeno-Lima S. Infection by Intestinal Parasites, Stunting and Anemia in School-Aged Children from Southern Angola. PloS one. 2015;10(9):e0137327. doi:10.1371/journal.pone.0137327
20. Fuglestad AJ, Lehmann AE, Kroupina MG, Petryk A, Miller BS, Iverson SL, et al. Iron deficiency in international adoptees from Eastern Europe. The Journal of pediatrics. 2008;153(2):272-7. doi:10.1016/j.jpeds.2008.02.048
21. Doom JR, Gunnar MR, Georgieff MK, Kroupina MG, Frenn K, Fuglestad AJ, et al. Beyond stimulus deprivation: iron deficiency and cognitive deficits in postinstitutionalized children. Child development. 2014;85(5):1805-12. doi:10.1111/cdev.12231
22. Fuglestad AJ, Georgieff MK, Iverson SL, Miller BS, Petryk A, Johnson DE, et al. Iron deficiency after arrival is associated with general cognitive and behavioral impairment in post-institutionalized children adopted from Eastern Europe. Maternal and child health journal. 2013;17(6):1080-7. doi:10.1007/s10995-012-1090-z
23. Doom JR, Georgieff MK, Gunnar MR. Institutional care and iron deficiency increase ADHD symptomology and lower IQ 2.5-5 years post-adoption. Developmental science. 2015;18(3):484-94. doi:10.1111/desc.12223
24. Dekker MC, Koot HM, van der Ende J, Verhulst FC. Emotional and behavioral problems in children and adolescents with and without intellectual disability. Journal of child psychology and psychiatry, and allied disciplines. 2002;43(8):1087-98.
25. Afrin T, Murase K, Kounosu A, Hunt VL, Bligh M, Maeda Y, et al. Sequential Changes in the Host Gut Microbiota During Infection With the Intestinal Parasitic Nematode Strongyloides venezuelensis. Frontiers in cellular and infection microbiology. 2019;9:217. doi:10.3389/fcimb.2019.00217
26. Beatty JK, Akierman SV, Motta JP, Muise S, Workentine ML, Harrison JJ, et al. Giardia duodenalis induces pathogenic dysbiosis of human intestinal microbiota biofilms. International journal for parasitology. 2017;47(6):311-26. doi:10.1016/j.ijpara.2016.11.010
27. Partida-Rodriguez O, Serrano-Vazquez A, Nieves-Ramirez ME, Moran P, Rojas L, Portillo T, et al. Human Intestinal Microbiota: Interaction Between Parasites and the Host Immune Response. Archives of medical research. 2017;48(8):690-700. doi:10.1016/j.arcmed.2017.11.015
28. Toro-Londono MA, Bedoya-Urrego K, Garcia-Montoya GM, Galvan-Diaz AL, Alzate JF. Intestinal parasitic infection alters bacterial gut microbiota in children. PeerJ. 2019;7:e6200. doi:10.7717/peerj.6200
29. Zaiss MM, Harris NL. Interactions between the intestinal microbiome and helminth parasites. Parasite immunology. 2016;38(1):5-11. doi:10.1111/pim.12274
30. Cenit MC, Sanz Y, Codoner-Franch P. Influence of gut microbiota on neuropsychiatric disorders. World journal of gastroenterology. 2017;23(30):5486-98. doi:10.3748/wjg.v23.i30.5486
31. Kim HN, Yun Y, Ryu S, Chang Y, Kwon MJ, Cho J, et al. Correlation between gut microbiota and personality in adults: A cross-sectional study. Brain, behavior, and immunity. 2018;69:374-85. doi:10.1016/j.bbi.2017.12.012
32. Bruch JD. Intestinal infection associated with future onset of an anxiety disorder: Results of a nationally representative study. Brain, behavior, and immunity. 2016;57:222-6. doi:10.1016/j.bbi.2016.05.014
33. Jiang H, Ling Z, Zhang Y, Mao H, Ma Z, Yin Y, et al. Altered fecal microbiota composition in patients with major depressive disorder. Brain, behavior, and immunity. 2015;48:186-94. doi:10.1016/j.bbi.2015.03.016
34. Ganci M, Suleyman E, Butt H, Ball M. The role of the brain-gut-microbiota axis in psychology: The importance of considering gut microbiota in the development,

perpetuation, and treatment of psychological disorders. Brain and behavior. 2019;9(11):e01408. doi:10.1002/brb3.1408

35. Raaska H, Elovainio M, Sinkkonen J, Matomaki J, Makipaa S, Lapinleimu H. Internationally adopted children in Finland: parental evaluations of symptoms of reactive attachment disorder and learning difficulties - FINADO study. Child: care, health and development. 2011;38(5):697-705. doi:10.1111/j.1365-2214.2011.01289.x

36. WHO Multicentre Growth Reference Study Group. WHO Child Growth Standards: Length/height-for-age, weight-for-age, weight-for-length, weight-for-height and body mass index-for-age: Methods and development. Geneva: World Health Organization. 2006.

37. Achenbach TM, & Rescorla, L.A. Manual for the ASEBA School-Age Forms & Profiles. University of Vermont, Research Center for Children, Youth & Families. 2001.

38. Ximenez C, Torres J. Development of Microbiota in Infants and its Role in Maturation of Gut Mucosa and Immune System. Archives of medical research. 2017;48(8):666-80. doi:10.1016/j.arcmed.2017.11.007

39. Gilliam WS, de Mesquita PB. The Relationship Between Language and Cognitive Development and Emotional ‐ Behavioral Problems in Financially ‐ Disadvantaged Preschoolers: A Longitudinal Investigation. Early Child Development and Care. 2000;162(1):9-24. doi:10.1080/0300443001620102

40. Steinberg L. Cognitive and affective development in adolescence. Trends Cogn Sci. 2005;9(2):69-74. doi:10.1016/j.tics.2004.12.005

41. Neuman H, Forsythe P, Uzan A, Avni O, Koren O. Antibiotics in early life: dysbiosis and the damage done. FEMS microbiology reviews. 2018;42(4):489-99. doi:10.1093/femsre/fuy018

42. Iizumi T, Battaglia T, Ruiz V, Perez Perez GI. Gut Microbiome and Antibiotics. Archives of medical research. 2017;48(8):727-34. doi:10.1016/j.arcmed.2017.11.004

43. Slykerman RF, Thompson J, Waldie KE, Murphy R, Wall C, Mitchell EA. Antibiotics in the first year of life and subsequent neurocognitive outcomes. Acta paediatrica (Oslo, Norway : 1992). 2017;106(1):87-94. doi:10.1111/apa.13613

44. Loughman A, Ponsonby AL, O'Hely M, Symeonides C, Collier F, Tang MLK, et al. Gut microbiota composition during infancy and subsequent behavioural outcomes. EBioMedicine. 2020;52:102640. doi:10.1016/j.ebiom.2020.102640

45. van Schaik R, Wolfs TF, Geelen SP. Improved general health of international adoptees, but immunization status still insufficient. European journal of pediatrics. 2009;168(9):1101-6. doi:10.1007/s00431-008-0895-7

46. Chiappini E, Bortone B, Borgi S, Sollai S, Matucci T, Galli L, et al. Infectious Diseases in Internationally Adopted Children and Intercountry Discrepancies Among Screening Protocols, A Narrative Review. Frontiers in pediatrics. 2019;7:448. doi:10.3389/fped.2019.00448

47. Ivanova MY, Dobrean A, Dopfner M, Erol N, Fombonne E, Fonseca AC, et al. Testing the 8-syndrome structure of the child behavior checklist in 30 societies. Journal of clinical child and adolescent psychology : the official journal for the Society of Clinical Child and Adolescent Psychology, American Psychological Association, Division 53. 2007;36(3):405-17. doi:10.1080/15374410701444363

Figure

Click here to download Figure Figure Intestinal parasites and behavioral problems_b.tif

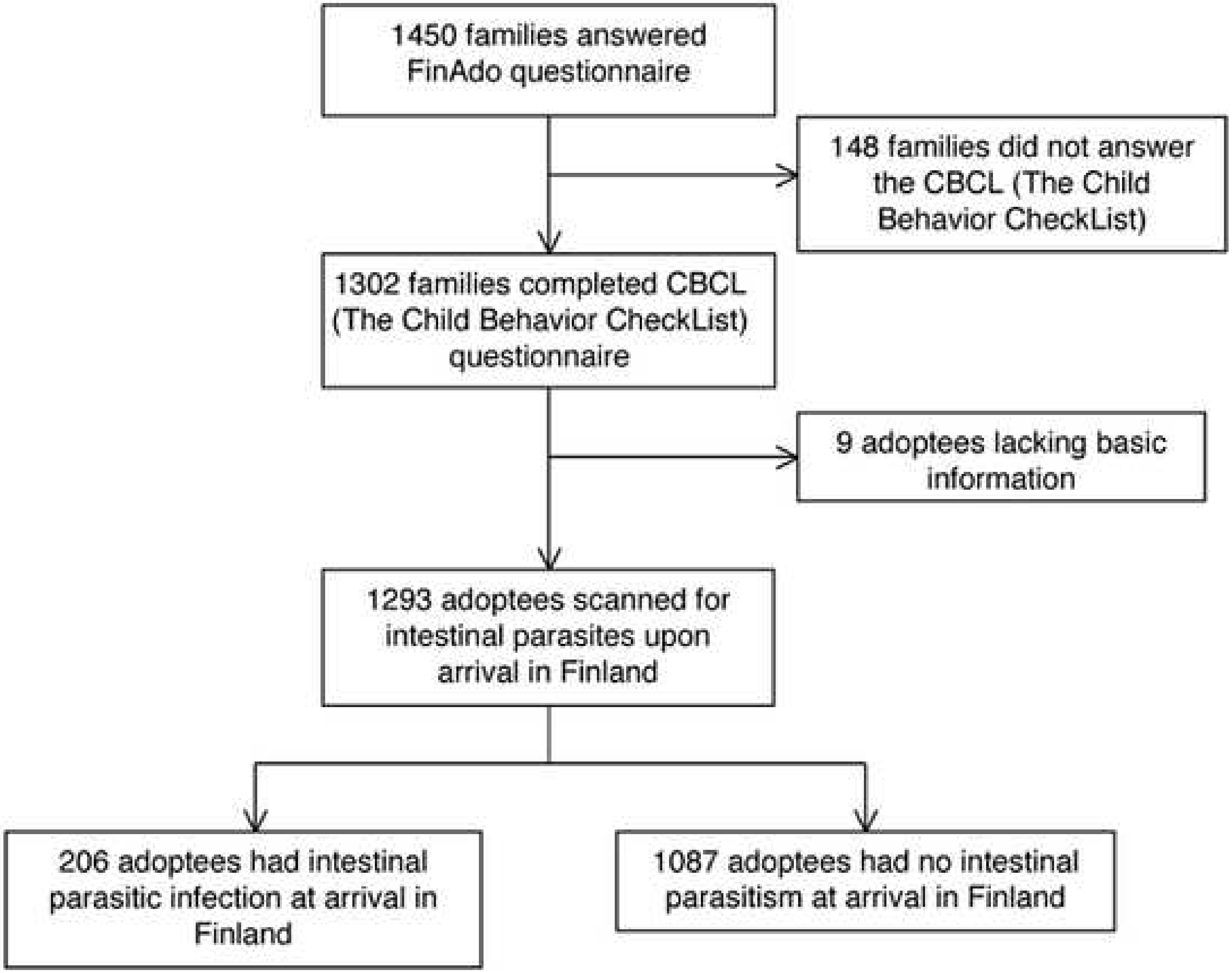

Supplement: Attachment — Submitted filename: reviewed PONE-D-20-08573.pdf [file pone.0245786.s002.pdf]
