# Supplementary material for: Intestinal parasites may be associated with later behavioral problems in internationally adopted children
Source: PLoS One. 2021 Jan 25;16(1):e0245786. doi: 10.1371/journal.pone.0245786 (PMC7833226; doi:10.1371/journal.pone.0245786)
Supplement: Attachment — Submitted filename: Response to Editor.docx [file pone.0245786.s004.docx]

Dear Dr. Dillman,

Thank you for your encouraging and valuable comments on our manuscript and the opportunity to revise it once more. We have now taken into account the latest remarks and have revised the manuscript accordingly.

Please find attached the revised version of the manuscript and the point-by-point responses to the comments.

We hope we have addressed all of your concerns and look forward to hearing from you.

Anna-Riitta Heikkilä on behalf of all the authors

Point-by-point responses:

**Academic Editor’s Comments:**

**In particular, while the revised version now includes a discussion of limitations and includes some caveats, I am concerned that the title and abstract might be over-interpreted. The limitations should be acknowledged in the abstract, and the title should be adjusted accordingly, to be less assertive title regarding the observed association discussed in the paper. The authors can decide how to do this, but one suggestion would be; "Intestinal parasites may be associated with later behavioral problems in internationally adopted children".**

Our response: We thank the Editor the proposal to change the title of the paper and we have made it as suggested: “Intestinal parasites may be associated with later behavioral problems in internationally adopted children”.

The abstract has now also been modified and the main limitations of the study have also been recognized in the abstract. The change is made in the abstract as follows, beginning from the methods paragraph (page 2, lines 33-50):

“**Methods:** Data for this study were sourced from the Finnish Adoption Study (FinAdo) based on parental questionnaires for all internationally adopted children under 18 years (*n* = 1450) who arrived in Finland from 1985 to 2007. A total of 1293 families provided sufficient information on the adoptee’s background, parasitic status on arrival, and behavioral symptoms at the median time of 5 years after arrival (mean age = 7.8 years). Behavioral and emotional disorders were evaluated with the Child Behavior Checklist (CBCL). Statistical analyses were conducted using linear regression.

**Results:** Of the 1293 families, parents of 206 adoptive children reported intestinal parasites in their adopted children on arrival. Parasite-infected children had subsequently higher CBCL problem scores than the children without parasites (*p* < 0.001). The association between intestinal parasites and later behavioral problems was stronger than that between intestinal parasites and any other factors measured in this study, except disability.

**Limitations:** The control group was naturally provided by the adopted children without parasite infections, but we could not compare the adopted children to non-adopted children without a defined parasite infection. We were unable to specify the effects associated with a specific parasite type. It was not possible either to include multiple environmental factors that could have been associated with behavioral problems in the models, which indicated only modest explanatory values.

**Conclusions:** In this study, intestinal parasite infections in early childhood may be associated with children’s later psychological wellbeing, even in children who move to a country with a low prevalence of parasites. Our findings may support further developments pertaining to the gut-brain theory.”
